# Does Sickle Cell Anaemia Have a Relationship With Avascular Pulp Necrosis? A Systematic Review

**DOI:** 10.1111/aej.70018

**Published:** 2025-09-08

**Authors:** Elidiane Elias Ribeiro, Karin Sá Fernandes, Erika Sales Joviano Pereira, Angelica Maria Cupertino Lopes Marinho, Jefferson R. Tenório, Márcia Pereira Alves dos Santos

**Affiliations:** ^1^ Department of Endodontics, School of Dentistry Universidade do Estado do Rio de Janeiro Rio de Janeiro Brazil; ^2^ Department of Dental Clinic, School of Dentistry Federal University of Rio de Janeiro Rio de Janeiro Brazil; ^3^ School of Dentistry Universidade Cidade de São Paulo São Paulo Brazil; ^4^ Department of Endodontics, School of Dentistry Universidade Federal da Bahia Salvador Bahia Brazil; ^5^ School of Dentistry – Universidade Federal de Minas Gerais Belo Horizonte Minas Gerais Brazil; ^6^ Department of Pathology and Oral Diagnosis, School of Dentistry Universidade Federal do Rio de Janeiro Rio de Janeiro Brazil; ^7^ Department of Forensic Dentistry and Public Health, School of Dentistry Universidade Federal do Rio de Janeiro Rio de Janeiro Brazil

**Keywords:** avascular necrosis, oral health, pulp necrosis, sickle cell anaemia, systematic review

## Abstract

This systematic review examined the relationship between sickle cell disease (SCD), an inherited genetic hemoglobinopathy, and avascular pulp necrosis (APN) in intact teeth. A comprehensive search of eight electronic databases was performed up to December 2024. Eligible studies included observational or interventional designs reporting APN in intact teeth of individuals with SCD. Risk of bias was assessed with the Joanna Briggs Institute Critical Appraisal Checklists, and certainty of evidence with GRADE. The search retrieved 407 records; four observational studies (two cohort and two case–control) met inclusion criteria, comprising 311 individuals with sickle cell anaemia (SCA). There is a relationship between SCA and APN. However, all included studies presented a risk of bias of 30% or higher, and the certainty of evidence was rated very low. These results provide weak scientific support for the association between APN and SCA, underscoring methodological limitations in the current dental literature.

## 1 Introduction

Inflammation and infections play a crucial role in the pathophysiology of sickle cell disease (SCD), driving both acute and chronic complications because they contribute to endothelial injury and vaso‐occlusion, leading to vaso‐occlusive crises (VOC) and other manifestations of the disease [1]. The accurate diagnosis of pulp conditions in individuals with SCD is crucial to evaluate the potential extent of inflammation and/or infection in the pulp tissue and to establish the suitable dental pulp intervention [2]. Pulpal disease typically arises from irreversible injury to the dental pulp due to significant stimuli, including dental caries, accidental trauma or iatrogenic interventions [3, 4]. However, in SCD, pulpal disease may also manifest in intact teeth [1]. Encased within rigid dentinal walls, the dental pulp is a highly vascularised and innervated tissue that performs the essential functions of nutrition, defence, and innervation of the teeth [5, 6]. The loss of this tissue compromises tooth vitality, leading to the diagnosis of pulp necrosis (PN), characterised by septic or aseptic breakdown of the pulpal connective tissue due to destruction of its microvascular, lymphatic, cellular and nervous fibre systems [7].

The absence of radiographic periapical lesions does not guarantee pulpal health, as pulp necrosis can occur without presenting clinical symptoms [8, 9]. Avascular (asymptomatic) pulp necrosis (APN) results from interruption of the vascular and nervous bundle at the apical foramen, characterised by protein denaturation, water and nutrient loss, leading to cellular degeneration and progression to a nonviable, coagulative state [10, 11, 12]. Notably, this condition can occur in intact teeth without exposure to the oral environment or periodontal involvement [2]. Also referred to as aseptic necrosis, this phenomenon affects other infarcted areas with low lysosomal activity, such as the dental pulp [10, 12].

SCD encompasses a set of autosomal recessive blood disorders characterised by the predominant presence of abnormal haemoglobin S associated with another haemoglobin variant resulting from a missense mutation (Glu6Val, rs334) in the β‐globin gene (HBB) [11]. This condition leads to the polymerisation of haemoglobin S, causing red blood cells to assume a sickle shape under hypoxic conditions, thereby impairing their function and reducing their lifespan [5]. Sickle cell anaemia (SCA) represents the most common genotype (Hb SS) of SCD and is clinically linked to an increased frequency and severity of pain crises and systemic complications [1, 4, 5, 6].

SCD is a multifaceted and globally prevalent condition observed in Europe, Africa, the United States, Central America, Asia and Brazil [1]. Due to its high morbidity and mortality rates, it is considered a global public health issue [13]. This benign hereditary haematological disorder is characterised by VOC and haemolytic anaemia, which can lead to oral alterations such as pale and jaundiced mucosa, delayed tooth eruption, neuropathies [12, 14], and mandibular condyle necrosis [15] and avascular pulp necrosis (APN), although these findings are not pathognomonic [16].

Avascular necrosis (AVN) is one of the consequences of VOC [17]. This process is attributed to red blood cell sickling and repetitive vaso‐occlusion, which are associated with tissue hypoxia, inflammation, and subsequent necrosis and bone collapse [18, 19]. In the pulpal tissue, the formation of a thrombus disrupts blood flow by blocking small blood vessels called arterioles. VOC affects the dental pulp, causing symptomatic or asymptomatic pulp necrosis even in the absence of odontogenic pathology in apparently healthy teeth [11]. Changes in blood flow, whether they are increased or decreased, can lead to irreversible tissue damage, such as calcification [20] or necrosis [11].

Individuals with SCD experiencing pain crises and dental infections are more likely to require hospitalisation and experience longer hospital stays compared with those without dental infections [21]. Therefore, we conducted this systematic review to better understand the relationship between APN and SCD, providing scientific evidence to guide the dental management of patients with this condition.

## 2 Methods

### 2.1 Protocol and Registration

The protocol for this systematic review was registered with the PROSPERO database (registration number CRD42020204171) and followed the Preferred Reporting Items for Systematic Review and Meta‐Analysis (PRISMA) recommendation (http://www.prisma‐statement.org) (Supporting Information 1).

### 2.2 Focused Question

APN [2] is one of the most frequently cited oral complications in the literature associated with SCD. However, there is no consensus regarding this relationship with SCD. This systematic literature review was conducted to address the question: Is there any relationship between APN and SCD? Two independent reviewers (E.E.R. and J.R.T.) conducted a systematic search in the electronic databases PubMed/MEDLINE, Scopus, Web of Science, Lilacs, Cochrane and Embase. Grey literature was also searched using Google Scholar and ProQuest. The initial search was conducted on 3 February 2024, in all languages and without any filters. The selection of descriptors was based on the most frequently cited terms in prior publications related to this topic. Boolean operators ‘AND’ and ‘OR’ were applied to develop the search strategy, in accordance with the syntax rules of each database (Supporting Information 2). A monthly search alert was set up, following the predefined search strategy, to notify the authors of any new studies until December 2024. To date, no additional studies meeting the eligibility criteria have been identified. Additionally, a careful manual examination of the reference lists of the studies included was conducted to prevent overlooking any pertinent publications. And finally, research in grey literature was also done.

### 2.3 Study Selection

#### 2.3.1 Inclusion Criteria

The inclusion criteria for this systematic review were established using the PECOs framework, as follows: P (population): humans; E (exposure): SCD; C (controlled group): healthy individuals (when available) or individuals with SCD without APN; O (outcome): presence of APN in individuals with teeth without caries, dental trauma, or iatrogenic interventions; s (study design): observational and/or interventional studies.

This systematic review aimed to include observational and interventional studies that investigated the occurrence of avascular dental pulp necrosis in patients with SCD without any restrictions on language or publication date.

The clinical inclusion criterion was studies with individuals with SCD (SCA, haemoglobin SC disease, haemoglobin SD disease, and S‐βThalassemias) who presented the teeth with a negative pulp response after applying a sensitive pulp test without any clinical signs such as dental caries, dental trauma or iatrogenic interventions. When a control group composed of individuals without SCD was included, these data were also extracted.

#### 2.3.2 Exclusion Criteria

The exclusion criteria involved case reports/series, literature reviews, animal studies, laboratory studies, book chapters, letters to the editor and conference/congress abstracts, so that these were also excluded.

The articles retrieved from the initial search were imported into EndNote X9 software (Thomson Reuters) for duplicate removal. Subsequently, the articles were exported to Rayyan (https://www.rayyan.ai). Titles and abstracts were independently analysed by two authors (E.E.R. and J.R.T.), and potential studies were thoroughly reviewed to determine their eligibility. In case of any discrepancy, a third co‐author (A.M.C.L.M.) was consulted to make the final decision. This pertains to the inclusion or exclusion of articles.

The clinical exclusion criterion was studies that included individuals with SCD who presented a negative pulp response and any clinical signs, such as dental caries, dental trauma or iatrogenic interventions in the teeth involved.

### 2.4 Data Extraction

The following data were collected: author and year of publication, country, study design, type of SCD, sample size (*n*), participants' age (mean and/or range), methodology (diagnosis methods) and main results (evaluation of APN).

### 2.5 Quality Assessment

The risk of bias was assessed independently by two reviewers (E.E.R. and E.S.J.P.) using the Joanna Briggs Institute (JBI) Critical Appraisal Checklists for Observational Studies. For studies, all eight items were applied. Each criterion was categorised as ‘Y’ (yes), ‘N’ (no) or ‘U’ (unclear) for risk of bias. An increasing number of ‘N’ and ‘U’ scores suggests a higher risk. Disagreements between the reviewers about the quality assessment were resolved by a third author (M.P.A.S.).

### 2.6 Grading of Evidence

The quality of evidence was evaluated with the Grading of Recommendations, Assessment, Development and Evaluation (GRADE) approach, employing the GRADE pro tool [22]. Risks of bias, inconsistency, indirectness, and imprecision were the items considered to rate the overall quality of evidence [22, 23]. All judgements were adapted to qualify the evidence synthesised in a narrative manner.

## 3 Results

### 3.1 Study Selection

**FIGURE 1:**
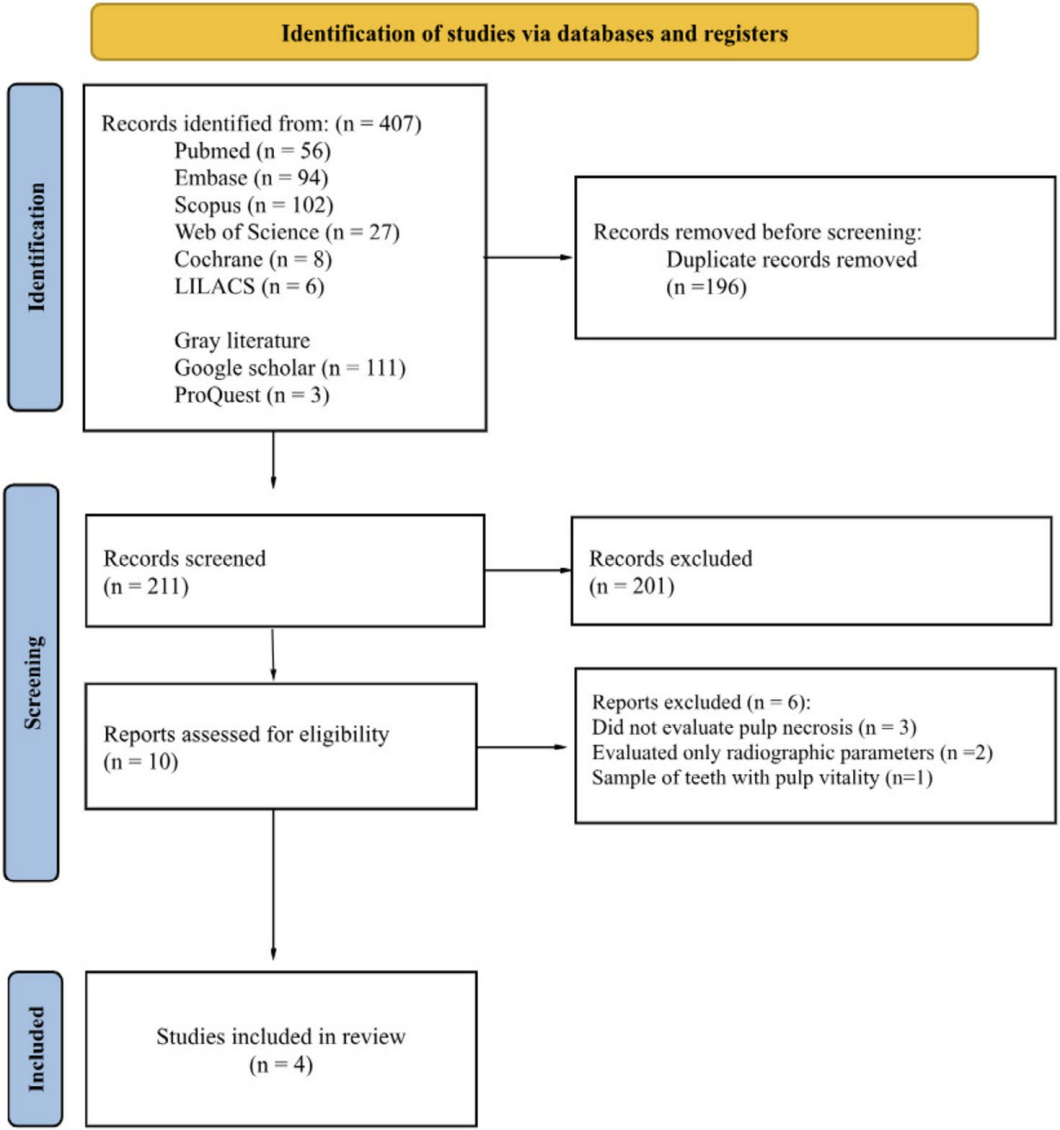
PRISMA flowchart.

In the initial search, 407 studies were identified (Figure 1). After removing duplicates, 211 studies were screened based on their titles and abstracts. Of these, 10 studies were deemed eligible and were reviewed in full. Following the application of the eligibility criteria, six studies [24, 25, 26, 27, 28, 29] were excluded following the full text review. Ultimately, four studies were included in this systematic review [30, 31, 32, 33].

### 3.2 Characteristics of Studies and Data Extraction

Four observational studies were included for data extraction: two are case–control [30, 31], and two are cohort [32, 33] (Table 1). Two studies were conducted in Brazil [32, 33], Turkey [31] and the USA [30]. All studies were conducted with individuals with SCA.

**TABLE 1:** General characteristics of the included studies.

Authors/country/year/study design	Participants			Diagnostic methods	Results	Presence of PN in individuals with SCA (proportion)	Main conclusions
	Sample size (N)	Groups	Age (mean ± SD or range in years)				
Andrews et al. (1983) USA Case–control^a^ [30]	22	SCA = 22 Control group = no data reported	12–37 years old	Clinical, radiographic, electric pulp, thermal, percussion and cavity tests	5 individuals with SCA and PN	23% (5/22)	PN is related to SCA due to compromise in the pulpal microcirculation.
Demirbaş Kaya et al. (2004) Turkey Case control [31]	72	SCA = 36 and 827 teeth (mean of 23 teeth per individual with SCA) Control group = 36 and 1084 teeth (mean of 30 teeth per individual)	16–40 years old	Clinical, Questionnaire, radiographic, thermal, percussion and cavity test.	51 teeth with PN in individuals with SCA 2,3 individuals with SCA and PN *p* < 0.05	6.4% (2.3/36)	PN is related to SCA because of compromised pulpal microcirculation, in healthy teeth.
Costa et al. (2013) Brazil Cohort [32]	339	SCA = 113 individuals and 113 teeth (mean of 1 tooth per individual with SCA) Control group = 226	> 16 years old	Clinical, thermal (hot/cold), percussion, and pulse oximetry.	11 teeth with PN Odds = 8.33 *p* = 0.05 11 individuals with SCA and NP	10% (11/113)	SCA is a potential risk factor for PN of clinically intact teeth. The evaluation of the pulp in clinically intact teeth should be routine in patients with SCA.
Costa et al. (2020) Brazil Cohort [33]	140	SCA = 140	> 16 years old	Clinical, thermal (hot/cold), percussion and pulse oximetry.	15 patients with pulp necrosis. *p* = 0.032	11% (15/140)	PN in permanent teeth in SCA individuals were associated with, osteoarticular injuries. PN can be considered an indicator of the lethality of the SCA.

Abbreviations: Hb SS, haemoglobin SS; PN, pulp necrosis; SCA, sickle cell anaemia; SCD, sickle cell disease.

^a^ As reported by the authors' manuscript.

The diagnostic methods for APN used in these studies included sensitivity tests (cold/hot), pulp oximetry adapted for dentistry, cavity tests and electric pulp tests. All studies used cold sensitivity tests and percussion, and two of them complemented these with hot sensitivity tests [32, 33]. Cavity tests without local anaesthesia for confirming APN were used in two studies [30, 31]. Periapical and panoramic radiographs and cone beam computed tomography (CBCT) images were analysed for diagnostic purposes. Two studies did not mention the use of periapical or panoramic radiographs [32, 33].

Two studies used the number of patients with ANP as the outcome, while the other two used the number of teeth with APN. We calculated the ratio of teeth diagnosed with ANP to the total number of teeth in individuals with SCA across the studies to standardise the data and determine the occurrence of ANP. We then multiplied the results by the total number of SCA individuals to determine the total number of SCD and PN individuals. Then, we obtained a ratio between individuals with NP and SCA and individuals with SCA (Table 1). Finally, we achieved a more precise measurement of the occurrence of APN in individuals with SCA, thereby enhancing the evaluation of ANP screening in these patients.

### 3.3 Results of Individual Studies

In the study by Andrews et al. [30] 22 individuals with SCA were evaluated, and pulp necrosis was diagnosed by means of the thermal, electrical and percussion tests. Five patients (22.7%) showed radiographic evidence of bone lesions in 21 areas, with 8 (38.1%) associated with tooth apices, while the remaining 13 (61.9%) lesions were not in the periapical region. One patient had five periapical lesions in intact teeth, which did not respond to thermal or electrical tests. The study found that the number of areas with bone rarefaction increased with the age of individuals. The suggestion was that pulp necrosis might occur due to blockage of the microcirculation of dental pulp.

Demirbaş Kaya et al. [31] evaluated 36 patients with SCA and a control group of 36 individuals without the disease by means of clinical and radiographic tests. Diagnostic tests for pulp necrosis were applied to 827 unrestored teeth. In the SCA group, 51 teeth (6%) did not respond to the tests. Orofacial pain of unknown origin was reported in 83% of patients with SCA. Radiographs showed deterioration in bone tissue quality, particularly in the mandible, in 67% of SCA patients. Radiographic changes, such as cortical thinning and irregularities in the mandible, were observed in 22% of SCA patients. The study concluded that due to vaso‐occlusion, SCA can affect pulpal microcirculation, leading to a higher incidence of pulp necrosis in healthy teeth.

Costa et al. [32] conducted a study with 113 patients with SCA and 226 controls, in which 2628 clinically healthy teeth from both groups were evaluated for pulp necrosis using diagnostic tests, pulp sensitivity tests, and pulse oximetry adapted for dentistry (PO) by three calibrated examiners (*κ* = 0.9). The exposed and non‐exposed groups were statistically similar regarding variables such as orofacial trauma, sex, age and socioeconomic class. Factors such as orofacial trauma and folic acid use were considered in the statistical analysis to adjust for potential confounding variables. The results showed that the occurrence of PN was 8.33 times higher in the SCA group compared with the control group (*p* < 0.001). Only 17 individuals (5%; 95% CI, 2.95–7.91) had at least one clinically intact permanent tooth with PN detected by both diagnostic methods, 11 (64.7%) of whom were in the exposed group. The prevalence of teeth with PN was 18.3% (*n* = 62) according to both methods, with 50 cases (80.64%) occurring in the SCA group. There was also a higher frequency of PN among clinically intact permanent teeth in the exposed group compared with the non‐exposed group (*p* = 0.005). The results suggested that pulp condition assessment in intact teeth should be routine practice in patients with SCA, as it was considered a potential risk factor for PN in clinically healthy teeth.

Costa et al. [33] evaluated 140 individuals, 125 without pulp necrosis and 15 with PN. A theoretical model was constructed to analyse the relationship between PN, sickle cell crises, oxygen saturation (SpO_2_), and comorbidities. No significant association was found between the number of sickle cell crises and PN (SFL = 0.127; *p* = 0.596). Body SpO_2_ was not related to PN (SFL = −0.102; *p* = 0.485). PN was associated with higher mortality rates in patients with SCA. The use of hydroxyurea was linked to lower rates of comorbidities (SFL = −0.315; *p* = 0.014), indicating its role in reducing SCA complications. The theoretical model used in the study explained 64.8% of PN variability, with comorbidities and age being significant factors. PN was significantly associated with comorbidities, particularly osteoarticular lesions (SFL = 1.115; *p* = 0.032). The prevalence of osteoarticular lesions and mortality rates was higher among patients with PN (*p* = 0.009 and *p* < 0.001, respectively). Thus, PN was identified as a potential indicator of SCA severity, highlighting the importance of stricter dental monitoring and management for these patients.

### 3.4 Risk of Bias

As shown in Tables 2 and 3, all studies presented a high risk of bias (> 25%), reflecting a low level of methodological quality. The studies included primarily failed to describe confounding factors. Information regarding medication use, socioeconomic factors, age range, frequency of blood transfusions, general health status and systemic comorbidities that could potentially affect the outcomes was not reported in most case–control and cohort studies. One of the cohort studies identified age, hydroxyurea use and socioeconomic class as confounding variables. However, no study specified strategies to mitigate these confounders. In case–control studies, one study highlighted issues regarding the definition and selection of the control group, as it failed to specify the controls clearly, merely mentioning that they did not have SCA without providing sufficient details, and study the selection criteria, suggesting potential selection bias. None of the case–control studies reported blinding of evaluators, introducing measurement bias.

**TABLE 2:** Risk of bias of the included studies as evaluated with the Joanna Briggs Institute Critical Appraisal Checklists for case control observational studies.

	Andrews et al. (1983) [30]	Demirbaş Kaya et al. (2004) [31]
Q1. Were the groups comparable other than the presence of disease in cases or the absence of disease in controls?	N	Y
Q2. Were cases and controls matched appropriately?	U	Y
Q3. Were the same criteria used for identification of cases and controls?	U	Y
Q4. Was exposure measured in a standard, valid and reliable way?	U	Y
Q5. Was exposure measured in the same way for cases and controls?	U	Y
Q6. Were confounding factors identified?	N	N
Q7. Were strategies to deal with confounding factors stated?	N	N
Q8. Were outcomes assessed in a standard, valid and reliable way for cases and controls?	N	Y
Q9. Was the exposure period of interest long enough to be meaningful?	U	U
Q10. Was appropriate statistical analysis used?	N	Y
Overall risk	100%	30%

*Note:* The number after each of the 10 items in the checklist indicates the number of studies receiving an assessment of ‘Not’ or ‘Unclear’. The overall risk of bias is calculated as the percentage of the 10 items in the checklist receiving an assessment of ‘Not’ or ‘Unclear’ for each of the 02 studies.

**TABLE 3:** Risk of bias of the included studies as evaluated with the Joanna Briggs Institute Critical Appraisal Checklists for cohort observational studies.

	Costa et al. (2013) [32]	Costa et al. (2020) [33]
Q1. Were the two groups similar and recruited from the same population?	Y	Y
Q2. Were the exposures measured similarly to assigning people to both exposed and unexposed groups?	Y	Y
Q3. Was the exposure measured in a valid and reliable way?	Y	U
Q4. Were confounding factors identified?	N	Y
Q5. Were strategies to deal with confounding factors stated?	N	U
Q6. Were the groups/participants free of the outcome at the start of the study (or at the moment of exposure)?	N	N
Q7. Were the outcomes measured in a valid and reliable way?	U	Y
Q8. Was the follow up time reported and sufficient to be long enough for outcomes to occur?	U	U
Q9. Was follow up complete, and if not, were the reasons to loss to follow up described and explored?	U	N
Q10. Were strategies to address incomplete follow up utilised?	N	Y
Q11. Was appropriate statistical analysis used?	Y	Y
Overall risk	63.63%	45.45%

*Note:* The number after each of the 11 items in the checklist indicates the number of studies receiving an assessment of ‘Not’ or ‘Unclear’. The overall risk of bias is calculated as the percentage of the 11 items in the checklist receiving an assessment of ‘Not’ or ‘Unclear’ for each of the 02 studies.

Another critical point was the lack of information regarding the use of periapical radiographs (PA) to confirm outcomes or exposures in the cohort studies. Although participant loss to follow‐up was reported, the authors did not specify how this issue was addressed, making it difficult to evaluate its impact on the results. Regarding the diagnosis of pulp necrosis, the cohort studies did not mention the use of radiographs as a complementary diagnostic tool. The use of radiographs to establish the diagnosis of APN in individuals with SCA is extremely important because APN can occur in teeth with crown integrity, without symptoms, which differs from dental trauma and/or dental caries. Additionally, radiographs can illustrate an inflammation or infection evolution process that acts as a stimulus of VOC.

Follow‐up duration was another critical issue, being absent in case–control studies and insufficiently described in cohort studies to ensure outcomes were adequately observed. Moreover, none of the studies included sociodemographic, clinical, or laboratory data that adequately characterised patients with SCA and their controls.

### 3.5 Quality of Evidence

The quality of the evidence for assessing the occurrence of APN in intact teeth of individuals with SCA, documented in Table 4, was classified as very low for both studies, whether the outcome was the number of teeth with pulp necrosis or the number of patients with pulp necrosis. Characteristics associated with inconsistency, such as overlapping test results between studies and indirectness, were considered non‐serious. The assessment of indirectness was considered non‐serious because, even without direct evidence, the exposure (SCD) and the outcome (APN) were adequately assessed by the main available instruments, allowing for comparison between studies. However, the included studies' design and the previously described methodological flaws reduced the overall level of evidence. Additionally, imprecision was categorised as grave since the presence of the outcome—in this case, dichotomous (presence/absence of pulp necrosis)—was observed in fewer than 300 events.

**TABLE 4:** Certainty of evidence.

Certainty assessment						No. of patients		Certainty
No. of studies	Study design	Risk of bias	Inconsistency	Indirectness	Imprecision	Sickle cell anaemia	Pulp necrosis	
Number of patients with pulp necrosis in individuals with sickle cell anaemia								
3	Non‐randomised studies	Very serious^a^	Not serious	Not serious	Very serious^b^	194	28	⨁◯◯◯
								Very low
Number of teeth with pulp necrosis in individuals with sickle cell anaemia								
1	Non‐randomised studies	Very serious^a^	Not serious	Not serious	Very serious^b^	36	51	⨁◯◯◯
								Very low

^a^ Most studies had a high risk of bias (≥ 70% NO responses).

^b^ Dichotomous outcome with < 300 events (pulp necrosis).

## 4 Discussion

This systematic review revealed that there is a relationship between individuals with SCA and the presence of PN in intact teeth, although there remains a need for strong scientific evidence. This relationship also occurs for other systemic diseases, such as cardiovascular disease, diabetes mellitus, liver disease, blood disorders and bone mineral density diseases that have already been associated with endodontic pathosis [34]. Furthermore, diabetes mellitus influenced the response of the pulp tissue and the pulp cells' behaviour, increasing the inflammation/degeneration in the pulp tissue while reducing cell proliferation [35]. It sounds interesting that pulps from individuals with diabetes mellitus have the tendency towards pulp necrosis caused by ischaemia [35] as seen for individuals with SCA, according to the studies reviewed.

Coagulative necrosis occurs when blood supply to a specific tissue is reduced or blocked, resulting in the coagulation of tissue proteins [12]. People with SCA may experience numerous complications due to the haemolysis of erythrocytes. Sickle‐shaped red blood cells gradually lose their ability to transport oxygen effectively, which can cause microcirculatory obstructions that lead to ischaemia and tissue inflammation; this results in painful crises, increased susceptibility to infections, and even organ damage [36]. These obstructions can occur within the pulp microcirculation, which may result in pulpal necrosis (PN) in otherwise intact teeth.

Although additional well‐designed clinical studies are needed, the results of this systematic review agree with previous findings reported and suggest that, in the absence of well‐designed observational controlled studies, the current evidence supports a relationship between APN and SCA. Furthermore, we found that APN affects approximately 13% of individuals with SCA on average. It demonstrated the importance of stricter dental monitoring and management for individuals with SCA, with clinical dentists and endodontists being advised about these conditions in their routine dental training programmes, and it brings one more contribution of this systematic review.

The American Association of Endodontists (AAE) establishes guidelines and standards for endodontic practice. Pulp sensitivity tests are essential tools for identifying pulp conditions [37]. All studies included in this review used at least one diagnostic method to evaluate pulp status. Proposed criteria for classifying non‐vital pulp include abscess/fistula, periapical bone rarefaction, inflammatory root resorption, interrupted root development, persistent crown discoloration, and loss of pulp sensitivity [38, 39]. Clinical examinations were conducted in all studies, while diagnostic imaging used in PN diagnosis included panoramic and PA in two studies [30, 31] and CBCT in two others [30, 31]. Radiography is an integral part of endodontic diagnosis [40]. PA are widely used to confirm periradicular bone loss near the periapical region [40]. However, PA has limitations, such as difficulty in detecting small changes in bone density [41]. Tomography provides greater precision and reliability in detecting periradicular changes compared with 2D images [41].

Diagnosing pulp status involves two approaches: pulp sensitivity and pulp vitality. Pulp sensitivity assesses the neural response, while pulp vitality evaluates the vascular response of the pulp. Thermal tests are simpler and more widely used for pulp diagnosis [42, 43, 44, 45, 46]. Common findings include a lack of response to stimuli, prolonged pain, or even exacerbated pain, which helps identify the tooth affected by pulp pathology [47]. Pulp sensitivity tests stimulate pulp nerve endings and include thermal (cold or hot) and electric pulp tests [48]. In this review, all studies used the cold test, with two studies also using the electric test for better diagnosis.

Studies [48, 49] have shown that PO is a promising alternative for pulp diagnosis. Despite its potential, PO presents challenges for use in the oral cavity and in reading SpO_2_ levels in dental structures [50]. A systematic review [51] analysed the diagnostic accuracy of pulp vitality and sensitivity tests and concluded that SpO_2_ demonstrated higher sensitivity and specificity than other methods, making it the most accurate test for determining pulp vitality. Two selected studies used SpO_2_ adapted for dentistry.

PN showed a significant association with comorbidities, particularly osteoarticular lesions, while the number of sickle cell crises and body SpO_2_ showed no statistical relevance. Furthermore, the mortality rate was significantly higher among patients with PN, suggesting that PN may indicate disease severity and a higher risk of mortality [33]. These findings demonstrate the importance of rigorous dental and multidisciplinary follow‐up for SCA patients to ensure early detection of severe complications. Conversely, a systematic review that demonstrated the effectiveness of diagnosing pulpitis indicated that the primary issue in pulp diagnostics is the lack of a reliable reference standard under clinical conditions [52]. The authors concluded that the most promising current approach appears to involve defining a combination of various clinical tests and symptoms, and that molecular diagnosis may be necessary in the future to determine the best possible strategy for clinically diagnosing true pulpal conditions [52]. Until then, our findings point out that individuals with SCA should have periodic dental consultations to monitor their oral health.

Hydroxyurea (HU) is a widely used disease‐modifying drug due to its promotion of a reduction in vaso‐occlusive episodes [43] and a decrease in early mortality risk [53]. However, only one study analysed HU use among participants [33]. This study reported lower comorbidity rates among HU users, supporting evidence that HU improves hematologic parameters [54], leading to better clinical outcomes and a reduction in crisis frequency and intensity [55], although it did not significantly affect PN incidence. Increasing the number of studies that involve a significant number of participants in this context helps us confirm the role of HU in ANP.

In addition, daily folic acid use was reported in cohort studies with ambiguous results. In univariate analysis, increased PN incidence was associated with daily folic acid use, but in multivariate models, folic acid emerged as a potential protective factor for PN [32]. This finding reinforces the importance of adopting preventive measures and further investigation.

The relationship between PN and age is a relevant aspect in dentistry, as the prevalence and incidence of PN can vary significantly throughout life. Studies by Costa et al. observed that the frequency of intact permanent teeth with PN decreases with age [32, 33]. Increased tooth loss with age in these patients better explains this finding. This hypothesis was confirmed in record analyses from the same referral centre as the cited studies, showing an increase in dental procedures with age, such as restorations, extractions, and periodontal and endodontic treatments, reducing the chances of intact teeth with PN in older patients [56].

AVN remains a severe and prevalent complication in individuals with SCA [17], frequently affecting the femoral and humeral heads, and occasionally involving the temporomandibular joint [57]. Its multifocal nature reinforces a systemic aetiology, often progressing silently until advanced structural collapse becomes evident [58, 59]. Bone infarction results from episodes of vaso‐occlusion, medullary ischemia and tissue hypoxia caused by sickled erythrocytes, ultimately leading to coagulative cell death [60]. These findings support the hypothesis that microcirculatory impairments in SCA are not restricted to skeletal structures and may also affect the dental pulp, particularly in the absence of trauma or caries, as occurred in PN.

Despite the observed relationship between SCA and PN, we must recognise there are limitations to this systematic review. Current evidence remains insufficient to establish a definitive relationship between SCA and NP in intact teeth. Most of the available data derive from observational studies with a high risk of bias and limited sample sizes. Another potential risk of bias that might lower the quality of available evidence is the lack of appropriate matching between study and control groups. Well‐designed prospective studies are needed to investigate the underlying mechanisms and prevalence of NP in individuals with SCD. Such studies should adopt standardised diagnostic protocols, explore potential genetic markers, and integrate advanced imaging techniques to ensure methodological rigour and enhance diagnostic accuracy.

## 5 Conclusion

None of the studies identified and analysed in this systematic review met high methodological standards. Although additional well‐designed clinical studies are needed, the results of this systematic review suggest that, in the absence of well‐designed observational controlled studies, the current evidence supports a relationship between APN and SCA. Therefore, future investigations are urgently needed and should incorporate more participants, sociodemographic, clinical, and laboratory data to allow for a more comprehensive understanding of this relationship. These studies must also rigorously address potential confounding factors, especially the use of medications that may influence the clinical outcomes of individuals with SCA. Despite the low level of evidence currently available, continued exploration of pulp necrosis in patients with SCA is essential for improving the diagnostic accuracy and therapeutic decision‐making.

## Author Contributions

*Study concepts*: Karin Sá Fernandes, Márcia Pereira Alves dos Santos. *Study design*: Elidiane Elias Ribeiro, Angelica Maria Cupertino Lopes Marinho, Márcia Pereira Alves dos Santos. *Data acquisition*: Elidiane Elias Ribeiro, Jefferson R. Tenório. *Data analysis and interpretation*: Elidiane Elias Ribeiro, Erika Sales Joviano Pereira, Angelica Maria Cupertino Lopes Marinho. *Manuscript preparation*: Elidiane Elias Ribeiro, Jefferson R. Tenório. *Manuscript editing*: Erika Sales Joviano Pereira, Angelica Maria Cupertino Lopes Marinho, Jefferson R. Tenório, Márcia Pereira Alves dos Santos. *Manuscript review*: Erika Sales Joviano Pereira, Angelica Maria Cupertino Lopes Marinho, Jefferson R. Tenório, Márcia Pereira Alves dos Santos.

## Disclosure

Authorship Declaration: All authors affirm that they have made substantial contributions to the conception or design of the work, or to the acquisition, analysis or interpretation of data; have been involved in drafting or critically revising the manuscript for important intellectual content; have approved the final version for publication; and accept full responsibility for the integrity and accuracy of the work.

## Conflicts of Interest

The authors declare no conflicts of interest.

## Supporting information


**Supporting Information 1.** The recommendation from the Preferred Reporting Items for Systematic Review and Meta‐Analysis (PRISMA).

**Supporting Information 2.** Search strategies.

## Data Availability

The authors have nothing to report.

## References

[1] F. B. Piel , M. H. Steinberg , and D. C. Rees , “Sickle Cell Disease,” New England Journal of Medicine 376, no. 2 (2017): 1561–1573. 28423290 10.1056/NEJMra1510865

[2] L. L. Hsu and J. Fan‐Hsu , “Evidence‐Based Dental Management in the New Era of Sickle Cell Disease: A Scoping Review,” Journal of the American Dental Association (1939) 151, no. 9 (2020): 668–677.e9. 32854869 10.1016/j.adaj.2020.05.023

[3] A. Jakovljevic , N. Nikolic , J. Jacimovic , et al., “Prevalence of Apical Periodontitis and Conventional Nonsurgical Root Canal Treatment in General Adult Population: An Updated Systematic Review and Meta‐Analysis of Cross‐Sectional Studies Published Between 2012 and 2020,” Journal of Endodontics 4610 (2020): 1371–1386. 10.1016/j.joen.2020.07.007 32673634

[4] C. S. Tibúrcio‐Machado , C. Michelon , F. B. Zanatta , M. S. Gomes , J. A. Marin , and C. A. Bier , “The Global Prevalence of Apical Periodontitis: A Systematic Review and Meta‐Analysis,” International Endodontic Journal 54 (2021): 712–735. 33378579 10.1111/iej.13467

[5] G. Schmalz , M. Widbiller , and K. M. Galler , “Clinical Perspectives of Pulp Regeneration,” Journal of Endodontics 46, no. 9 (2020): S161–S174. 32950188 10.1016/j.joen.2020.06.037

[6] E. Couve , M. Lovera , K. Suzuki , and O. Schmachtenberg , “Schwann Cell Phenotype Changes in Aging Human Dental Pulp,” Journal of Dental Research 97, no. 3 (2018): 347–355. 28972819 10.1177/0022034517733967

[7] M. Goldberg , A. Njeh , and E. Uzunoglu , “Is Pulp Inflammation a Prerequisite for Pulp Healing and Regeneration?,” Mediators of Inflammation 2015 (2015): 347649. 26538825 10.1155/2015/347649 PMC4619968

[8] A. Petersson , S. Axelsson , T. Davidson , et al., “Radiological Diagnosis of Periapical Bone Tissue Lesions in Endodontics: A Systematic Review,” International Endodontic Journal 45 (2012): 783–801. 22429152 10.1111/j.1365-2591.2012.02034.x

[9] P. L. Michaelson and G. R. Holland , “Is Pulpitis Painful?,” International Endodontic Journal 35 (2002): 829–832. 12406376 10.1046/j.1365-2591.2002.00579.x

[10] A. Consolaro and V. d. R. Bernardini , “Metamorfose cálcica da polpa e necrose pulpar asséptica no planejamento ortodôntico,” Revista Dental Press de Ortodontia e Ortopedia Facial 12 (2007): 21–23.

[11] J. E. Hasler and D. F. Mitchell , “Painless pulpitis,” Journal of the American Dental Association (1939) 81, no. 3 (1970): 671–677. 5271058 10.14219/jada.archive.1970.0293

[12] M. Kakkar , K. Holderle , M. Sheth , S. Arany , L. Schiff , and A. Planerova , “Orofacial Manifestation and Dental Management of Sickle Cell Disease: A Scoping Review,” Anemia 2021 (2021): 5556708. 34721900 10.1155/2021/5556708 PMC8556080

[13] T. Vos , S. S. Lim , C. Abbafati , et al., “Global Burden of 369 Diseases and Injuries in 204 Countries and Territories, 1990–2019: A Systematic Analysis for the Global Burden of Disease Study 2019,” Lancet 396 (2020): 1204–1222. 33069326 10.1016/S0140-6736(20)30925-9 PMC7567026

[14] N. Kawar , S. Alrayyes , and H. Aljewari , “Sickle Cell Disease: An Overview of Orofacial and Dental Manifestations,” Disease‐a‐Month 64 (2018): 290–295. 29338872 10.1016/j.disamonth.2017.12.004

[15] H. Yue , X. Xu , Q. Liu , X. Li , W. Jiang , and B. Hu , “Association Between Sickle Cell Disease and Dental Caries: A Systematic Review and Meta‐Analysis,” Hematology 25 (2020): 309–319. 32783601 10.1080/16078454.2020.1748927

[16] M. Chekroun , H. Chérifi , B. Fournier , et al., “Oral Manifestations of Sickle Cell Disease,” British Dental Journal 226 (2019): 27–31. 30631169 10.1038/sj.bdj.2019.4

[17] M. P. Leandro , N. D. Almeida , L. S. Hocevar , C. K. C. de Sá , A. J. Souza , and M. A. Matos , “Polymorphisms and Avascular Necrosis in Patients With Sickle Cell Disease—A Systematic Review,” Revista Paulista de Pediatria 40 (2022): e2021013. 35584416 10.1590/1984-0462/2022/40/2021013IN PMC9113627

[18] A. J. Martí‐Carvajal , I. Solà , and L. H. Agreda‐Pérez , “Treatment for Avascular Necrosis of Bone in People With Sickle Cell Disease,” Cochrane Database of Systematic Reviews 8 (2016): CD004344. 10.1002/14651858.CD004344.pub6 27502327

[19] Z. A. Naseer , M. Bachabi , L. C. Jones , R. S. Sterling , and H. S. Khanuja , “Osteonecrosis in Sickle Cell Disease,” Southern Medical Journal 109 (2016): 525–530. 27598354 10.14423/SMJ.0000000000000516

[20] N. N. Soni , “Microradiographic Study of Dental Tissues in Sickle‐Cell Anaemia,” Archives of Oral Biology 11, no. 6 (1966): 561–564. 5225861 10.1016/0003-9969(66)90221-4

[21] B. Laurence , C. Haywood , and S. Lanzkron , “Dental Infections Increase the Likelihood of Hospital Admissions Among Adult Patients With Sickle Cell Disease,” Community Dental Health 30 (2013): 168–172. 24151791 PMC4115243

[22] H. J. Schünemann , A. D. Oxman , J. Brozek , et al., “Grading Quality of Evidence and Strength of Recommendations for Diagnostic Tests and Strategies,” BMJ 336 (2008): 1106–1110. 18483053 10.1136/bmj.39500.677199.AE PMC2386626

[23] H. Balshem , M. Helfand , H. J. Schünemann , et al., “GRADE Guidelines: 3. Rating the Quality of Evidence,” Journal of Clinical Epidemiology 64 (2011): 401–406. 21208779 10.1016/j.jclinepi.2010.07.015

[24] H. L. C. C. de Carvalho , J. Y. S. Rolim , É. B. A. F. Thomaz , and S. F. C. Souza , “Are Dental and Jaw Changes More Prevalent in a Brazilian Population With Sickle Cell Anemia?,” Oral Surgery, Oral Medicine, Oral Pathology, Oral Radiology 124 (2017): 76–84. 28412236 10.1016/j.oooo.2017.02.016

[25] O. Felemban , L. Alhalees , L. Alattas , K. Baghlaf , and M. Aldajani , “Association Between Dental Infection and Increased Incidence of Complications in Sickle Cell Disease Children: A Cross‐Sectional Study in Jeddah City,” Journal of Advanced Oral Research 14 (2023): 54–60.

[26] L. M. E. Gibaly , M. Z. Radwan , A. M. Abdel‐Aziz , and G. Y. El Kamah , “Comparison of Two Vital Pulp Therapies in β‐Thalassemic Children,” Contemporary Pediatric Dentistry 1 (2020): 33–41.

[27] S. A. Costa , C. C. C. Ribeiro , E. B. A. F. Thomaz , C. P. S. Costa , and S. F. C. Souza , “Mechanisms Underlying the Adaptive Pulp and Jaw Bone Trabecular Changes in Sickle Cell Anemia,” Oral Diseases 29 (2023): 786–795. 34369045 10.1111/odi.13998

[28] S. B. Ferreira , W. L. Tavares , M. A. Rosa , et al., “Sickle Cell Anemia in Brazil: Personal, Medical and Endodontic Patterns,” Brazilian Oral Research 30 (2016): S1806‐83242016000100255. 10.1590/1807-3107BOR-2016.vol30.0060 27223130

[29] S. F. C. Souza , E. B. A. F. Thomaz , and C. P. S. Costa , “Healthy Dental Pulp Oxygen Saturation Rates in Subjects With Homozygous Sickle Cell Anemia: A Cross‐Sectional Study Nested in a Cohort,” Journal of Endodontics 43 (2017): 1997–2000. 29032814 10.1016/j.joen.2017.07.011

[30] C. H. Andrews , M. C. England , and W. B. Kemp , “Sickle Cell Anemia: An Etiological Factor in Pulpal Necrosis,” Journal of Endodontics 9 (1983): 249–252. 6579178 10.1016/s0099-2399(86)80023-1

[31] A. Demirbaş Kaya , B. O. Aktener , and C. Unsal , “Pulpal Necrosis With Sickle Cell Anaemia,” International Endodontic Journal 37 (2004): 602–606. 15317563 10.1111/j.1365-2591.2004.00853.x

[32] C. P. S. Costa , E. B. A. F. Thomaz , and S. F. C. Souza , “Association Between Sickle Cell Anemia and Pulp Necrosis,” Journal of Endodontics 39 (2013): 177–181. 23321227 10.1016/j.joen.2012.10.024

[33] C. P. S. Costa , É. B. A. F. Thomaz , C. C. C. Ribeiro , and S. F. C. Souza , “Biological Factors Associating Pulp Necrosis and Sickle Cell Anemia,” Oral Diseases 26 (2020): 1558–1565. 32413162 10.1111/odi.13415

[34] N. Khalighinejad , M. R. Aminoshariae , A. Aminoshariae , J. C. Kulild , A. Mickel , and A. F. Fouad , “Association Between Systemic Diseases and Apical Periodontitis,” Journal of Endodontics 42, no. 10 (2016): 1427–1434, 10.1016/j.joen.2016.07.007. 27592295

[35] R. M. N. Pimenta , A. H. Dos Reis‐Prado , S. de Castro Oliveira , et al., “Effects of Diabetes Mellitus on Dental Pulp: A Systematic Review of In Vivo and In Vitro Studies,” Oral Diseases 30, no. 2 (2024): 100–115, 10.1111/odi.14267. 35657117

[36] C. M. I. Lopes , S. S. Lira , J. C. da Silva Oliveira , et al., “Occlusal Disorders in Patients With Sickle Cell Disease: Critical Literature Review,” Journal of Clinical Pediatric Dentistry 45 (2021): 117–122. 33951171 10.17796/1053-4625-45.2.8

[37] I. K. Dhillon , C. H. L. Hong , S. Hu , et al., “Accuracy of the American Association of Endodontists Diagnostic Criteria for Assessing Pulp Health in Primary Teeth,” Clinical Oral Investigations 27 (2023): 6043–6053. 37624522 10.1007/s00784-023-05217-6

[38] F. M. Andreasen , “Pulpal Healing After Luxation Injuries and Root Fracture in the Permanent Dentition,” Endodontics & Dental Traumatology 5 (1989): 111–131. 2699588 10.1111/j.1600-9657.1989.tb00348.x

[39] I. Jacobsen , “Criteria for Diagnosis of Pulp Necrosis in Traumatized Permanent Incisors,” Scandinavian Journal of Dental Research 88 (1980): 306–312. 6934614 10.1111/j.1600-0722.1980.tb01231.x

[40] F. C. Setzer and S. M. Lee , “Radiology in Endodontics,” Dental Clinics of North America 65, no. 3 (2021): 475–486. 34051926 10.1016/j.cden.2021.02.004

[41] D. K. Rechenberg , A. Munir , and M. Zehnder , “Correlation Between the Clinically Diagnosed Inflammatory Process and Periapical Index Scores in Severely Painful Endodontically Involved Teeth,” International Endodontic Journal 54 (2021): 172–180. 32918280 10.1111/iej.13407 PMC7894281

[42] S. Patel , A. Dawood , E. Whaites , and T. Pitt Ford , “New Dimensions in Endodontic Imaging: Part 1. Conventional and Alternative Radiographic Systems,” International Endodontic Journal 42 (2009): 447–462. 19298577 10.1111/j.1365-2591.2008.01530.x

[43] R. A. Alghaithy and A. J. Qualtrough , “Pulp Sensibility and Vitality Tests for Diagnosing Pulpal Health in Permanent Teeth: A Critical Review,” International Endodontic Journal 50 (2017): 135–142. 26789282 10.1111/iej.12611

[44] M. Zanini , E. Meyer , and S. Simon , “Pulp Inflammation Diagnosis From Clinical to Inflammatory Mediators: A Systematic Review,” Journal of Endodontics 43 (2017): 1033–1051. 28527838 10.1016/j.joen.2017.02.009

[45] E. Chen and P. V. Abbott , “Dental Pulp Testing: A Review,” International Journal of Dentistry 2009 (2009): 365785. 20339575 10.1155/2009/365785 PMC2837315

[46] H. Jafarzadeh and P. V. Abbott , “Review of Pulp Sensibility Tests. Part I: General Information and Thermal Tests,” International Endodontic Journal 43 (2010): 738–762. 20609022 10.1111/j.1365-2591.2010.01754.x

[47] S. Patro , A. Meto , A. Mohanty , et al., “Diagnostic Accuracy of Pulp Vitality Tests and Pulp Sensibility Tests for Assessing Pulpal Health in Permanent Teeth: A Systematic Review and Meta‐Analysis,” International Journal of Environmental Research and Public Health 19 (2022): 9599. 35954958 10.3390/ijerph19159599 PMC9367848

[48] A. Mainkar and S. G. Kim , “Diagnostic Accuracy of 5 Dental Pulp Tests: A Systematic Review and Meta‐Analysis,” Journal of Endodontics 44, no. 5 (2018): 694–702. 29571914 10.1016/j.joen.2018.01.021

[49] C. L. Caldeira , F. B. Barletta , M. C. Ilha , C. V. Abrão , and G. Gavini , “Pulse Oximetry: A Useful Test for Evaluating Pulp Vitality in Traumatized Teeth,” Dental Traumatology: Official Publication of International Association for Dental Traumatology 32, no. 5 (2016): 385–409. 27140332 10.1111/edt.12279

[50] E. Calil , C. L. Caldeira , G. Gavini , and E. M. Lemos , “Determination of Pulp Vitality In Vivo With Pulse Oximetry,” International Endodontic Journal 41 (2008): 741–746. 18554185 10.1111/j.1365-2591.2008.01421.x

[51] T. Tamura , Y. Maeda , M. Sekine , and M. Yoshida , “Wearable Photoplethysmographic Sensors—Past and Present,” Electronics 3 (2014): 282–302.

[52] D. Donnermeyer , T. Dammaschke , M. Lipski , and E. Schäfer , “Effectiveness of Diagnosing Pulpitis: A Systematic Review,” International Endodontic Journal 56, no. Suppl 3 (2023): 296–325, 10.1111/iej.13762. 35536159

[53] M. Yang , L. Elmuti , and S. M. Badawy , “Health‐Related Quality of Life and Adherence to Hydroxyurea and Other Disease‐Modifying Therapies Among Individuals With Sickle Cell Disease: A Systematic Review,” BioMed Research International 2022 (2022): 2122056. 35898672 10.1155/2022/2122056 PMC9313963

[54] J. F. Tisdale , S. L. Thein , and W. A. Eaton , “Treating sickle cell anemia,” Science 367 (2020): 1198–1199. 32165573 10.1126/science.aba3827 PMC7299198

[55] S. Lanzkron , J. J. Strouse , R. Wilson , et al., “Systematic Review: Hydroxyurea for the Treatment of Adults With Sickle Cell Disease,” Annals of Internal Medicine 148 (2008): 939–955. 18458272 10.7326/0003-4819-148-12-200806170-00221 PMC3256736

[56] R. D. Cançado and J. A. Jesus , “A doença falciforme no Brasil,” Revista Brasileira de Hematologia e Hemoterapia 29 (2007): 204–206.

[57] C. P. S. Costa , B. T. C. Aires , E. B. A. F. Thomaz , and S. d. F. C. Souza , “Dental Care Provided to Sickle Cell Anemia Patients Stratified by Age: A Population‐Based Study in Northeastern Brazil,” European Journal of Dentistry 10 (2016): 356–360. 27403053 10.4103/1305-7456.184149 PMC4926588

[58] A. J. Martí‐Carvajal , I. Solà , and L. H. Agreda‐Pérez , “Treatment for Avascular Necrosis of Bone in People With Sickle Cell Disease,” Cochrane Database of Systematic Reviews 12, no. 12 (2019): CD004344. 31803937 10.1002/14651858.CD004344.pub7 PMC6894369

[59] H. E. Ware , A. P. Brooks , R. Toye , and S. I. Berney , “Sickle Cell Disease and Silent Avascular Necrosis of the Hip,” Journal of Bone and Joint Surgery. British Volume 73 (1991): 947–949, 10.1302/0301-620X.73B6.1955442. 1955442

[60] E. Bedair , N. Almaslamani , and M. Yassin , “Radiological Manifestation of Avascular Necrosis (AVN) in Sickle Cell Disease (SCD): A Review of Diagnostic Imaging,” Acta Bio‐Medica 94, no. 3 (2023): e2023177. 37326259 10.23750/abm.v94i3.14714 PMC10308460

