# Supplementary material for: Does Sickle Cell Anaemia Have a Relationship With Avascular Pulp Necrosis? A Systematic Review
Source: Aust Endod J. 2025 Sep 8;52(1):242–52. doi: 10.1111/aej.70018 (PMC13051008; doi:10.1111/aej.70018)
Supplement: Supplementary file 2 — Supporting Information 2. Search strategies. [file AEJ-52-242-s002.docx]

**Supplementary II** – Search strategies.

| **Database** | **Search strategy** |
| --- | --- |
| PubMed/Medline | ("Anemia, Sickle Cell"[Mesh] OR "Sickle Cell Anemia" OR "Sickle Cell Anemias" OR "Sickle Cell Anemia" OR "Sickle Cell Anemias" OR  "Sickle Cell Disease" OR "Sickle Cell Diseases" OR "Hemoglobin S Disease" OR "Hemoglobin S Diseases" OR "Sickle Cell Disorders" OR "Sickle Cell Disorder" OR "HbS Disease" OR "Sickle Hemoglobin" OR "Sickle cell Hemoglobin" OR "Hemoglobins"[Mesh:NoExp] OR "Hemoglobins"  OR "Hemoglobins" OR "Hemoglobins, Abnormal"[Mesh]  OR "Abnormal Hemoglobins" OR "Hemoglobin, Sickle"[Mesh]  OR "Sickle Hemoglobin" OR "hemoglobin S" OR "haemoglobin S" OR "hemoglobin SC" OR "haemoglobin SC" OR "hemoglobin SE" OR "haemoglobin SE" OR "hemoglobin SS" OR "haemoglobin SS" OR "hemoglobin C disease" OR "hemoglobin D disease" OR "hemoglobin E disease" OR "haemoglobin C disease" OR "haemoglobin D disease" OR "haemoglobin E disease" OR "Hb SC" OR HbSC OR HbAS OR HbSS OR HbAC OR "Hb SE" OR "Hb SS" OR "Hb C disease" OR "Hb D disease" OR "Hb E disease" OR "SC disease" OR "SC diseases" OR "sickle cell*" OR sicklemia OR sickled OR sickling OR meniscocyt* OR drepanocyt* OR "Hemoglobin SC Disease"[Mesh] OR "Hemoglobin SC Disease" OR "Hemoglobin SC Diseases" OR "Sickle Cell Hemoglobin C Disease" OR "Sickle Cell Trait"[Mesh] OR "Sickle Cell Trait" OR "Sickle Cell Traits" OR "Thalassemia"[Mesh:NoExp] OR "Thalassemi*" OR "alpha-Thalassemia"[Mesh] OR "beta-Thalassemia"[Mesh] OR "Mediterranean Anemia" OR "Erythrocyte Indices"[Mesh] OR "Erythrocyte Indices") AND ("Dental Pulp Necrosis"[Mesh] OR "Dental Pulp Necrosis" OR "Pulp Necrosis" OR "Pulpal Necrosis" OR "Pulpal Diagnosis of Necrosis" OR "Pulp Necroses" OR "Pulpal Necroses" OR "Pulpal Diagnosis of Necroses" OR "Pulp Mummification" OR "Pulp Mummifications" OR "Pulp Gangrene" OR "Pulp Gangrenes" OR "Dental Pulp"[Mesh] OR "Dental Pulp" OR "Dental Pulps" OR "Dental Pulp Test"[Mesh] OR "Dental Pulp Test" OR "Dental Pulp Diseases"[Mesh] OR "Dental Pulp Diseases" OR "Pulp Diseases" OR "Dental Pulp Devitalization"[Mesh] OR "Dental Pulp Devitalization" OR "Pulp Devitalization" OR "Dental Pulp Calcification"[Mesh] OR "Dental Pulp Calcification" OR "Pulp Calcification" OR "necrotic pulp") |
| Embase | ('anemia, sickle cell'/exp OR 'anemia, sickle cell' OR 'sickle cell anemia'/exp OR 'sickle cell anemia' OR 'sickle cell anemias' OR 'sickle cell anemia'/exp OR 'sickle cell anaemia' OR 'sickle cell anaemias' OR 'sickle cell disease'/exp OR 'sickle cell disease' OR 'sickle cell diseases' OR 'hemoglobin s disease' OR 'hemoglobin s diseases' OR 'sickle cell disorders' OR 'sickle cell disorder' OR 'hbs disease' OR 'sickle cell hemoglobin'/exp OR 'sickle cell hemoglobin' OR 'hemoglobins'/exp OR 'hemoglobins' OR 'haemoglobins'/exp OR 'haemoglobins' OR 'hemoglobins, abnormal'/exp OR 'hemoglobins, abnormal' OR 'abnormal hemoglobins' OR 'hemoglobin, sickle'/exp OR 'hemoglobin, sickle' OR 'sickle hemoglobin'/exp OR 'sickle hemoglobin' OR 'hemoglobin s'/exp OR 'hemoglobin s' OR 'haemoglobin s'/exp OR 'haemoglobin s' OR 'hemoglobin sc'/exp OR 'hemoglobin sc' OR 'haemoglobin sc'/exp OR 'haemoglobin sc' OR 'hemoglobin se' OR 'haemoglobin se' OR 'hemoglobin ss'/exp OR 'hemoglobin ss' OR 'haemoglobin ss'/exp OR 'haemoglobin ss' OR 'hemoglobin c disease'/exp OR 'hemoglobin c disease' OR 'hemoglobin d disease'/exp OR 'hemoglobin d disease' OR 'hemoglobin e disease'/exp OR 'hemoglobin e disease' OR 'haemoglobin c disease'/exp OR 'haemoglobin c disease' OR 'haemoglobin d disease'/exp OR 'haemoglobin d disease' OR 'haemoglobin e disease' OR 'hb sc' OR hbsc OR hbas OR hbss OR hbac OR 'hb se' OR 'hb ss' OR 'hb c disease'/exp OR 'hb c disease' OR 'hb d disease'/exp OR 'hb d disease' OR 'hb e disease' OR 'sc disease' OR 'sc diseases' OR 'sickle cell*' OR sicklemia OR sickled OR sickling OR meniscocyt* OR drepanocyt* OR 'hemoglobin sc disease'/exp OR 'hemoglobin sc disease' OR 'hemoglobin sc diseases' OR 'sickle cell hemoglobin c disease'/exp OR 'sickle cell hemoglobin c disease' OR 'sickle cell trait'/exp OR 'sickle cell trait' OR 'sickle cell traits' OR 'thalassemia'/exp OR 'thalassemia' OR 'thalassemi*' OR 'alpha-thalassemia'/exp OR 'alpha-thalassemia' OR 'beta-thalassemia'/exp OR 'beta-thalassemia' OR 'mediterranean anemia'/exp OR 'mediterranean anemia' OR 'erythrocyte indices'/exp OR 'erythrocyte indices') AND ('dental pulp necrosis'/exp OR 'dental pulp necrosis' OR 'pulp necrosis' OR 'pulpal necrosis' OR 'pulpal diagnosis of necrosis' OR 'pulp necroses' OR 'pulpal necroses' OR 'pulpal diagnosis of necroses' OR 'pulp mummification' OR 'pulp mummifications' OR 'pulp gangrene' OR 'pulp gangrenes' OR 'dental pulp'/exp OR 'dental pulp' OR 'dental pulps' OR 'dental pulp test'/exp OR 'dental pulp test' OR 'dental pulp diseases'/exp OR 'dental pulp diseases' OR 'pulp diseases' OR 'dental pulp devitalization'/exp OR 'dental pulp devitalization' OR 'pulp devitalization'/exp OR 'pulp devitalization' OR 'dental pulp calcification'/exp OR 'dental pulp calcification' OR 'pulp calcification' OR 'necrotic pulp') |
| SCOPUS | TITLE-ABS-KEY("Sickle Cell Anemia" OR "Sickle Cell Anemias" OR "Sickle Cell Anaemia" OR "Sickle Cell Anaemias" OR  "Sickle Cell Disease" OR "Sickle Cell Diseases" OR "Hemoglobin S Disease" OR "Hemoglobin S Diseases" OR "Sickle Cell Disorders" OR "Sickle Cell Disorder" OR "HbS Disease" OR "Sickle Hemoglobin" OR "Sickle cell Hemoglobin" OR "Hemoglobins"  OR "Haemoglobins" OR "Abnormal Hemoglobins" OR "Sickle Hemoglobin" OR "hemoglobin S" OR "haemoglobin S" OR "hemoglobin SC" OR "haemoglobin SC" OR "hemoglobin SE" OR "haemoglobin SE" OR "hemoglobin SS" OR "haemoglobin SS" OR "hemoglobin C disease" OR "hemoglobin D disease" OR "hemoglobin E disease" OR "haemoglobin C disease" OR "haemoglobin D disease" OR "haemoglobin E disease" OR "Hb SC" OR HbSC OR HbAS OR HbSS OR HbAC OR "Hb SE" OR "Hb SS" OR "Hb C disease" OR "Hb D disease" OR "Hb E disease" OR "SC disease" OR "SC diseases" OR "sickle cell*" OR sicklemia OR sickled OR sickling OR meniscocyt* OR drepanocyt* OR "Hemoglobin SC Disease" OR "Hemoglobin SC Diseases" OR "Sickle Cell Hemoglobin C Disease" OR "Sickle Cell Trait" OR "Sickle Cell Traits" OR Thalassemi* OR "alpha-Thalassemia" OR "beta-Thalassemia" OR "Mediterranean Anemia" OR "Erythrocyte Indices") AND TITLE-ABS-KEY("Dental Pulp Necrosis" OR "Pulp Necrosis" OR "Pulpal Necrosis" OR "Pulpal Diagnosis of Necrosis" OR "Pulp Necroses" OR "Pulpal Necroses" OR "Pulpal Diagnosis of Necroses" OR "Pulp Mummification" OR "Pulp Mummifications" OR "Pulp Gangrene" OR "Pulp Gangrenes" OR "Dental Pulp" OR "Dental Pulps" OR "Dental Pulp Test" OR "Dental Pulp Diseases" OR "Pulp Diseases" OR "Dental Pulp Devitalization" OR "Pulp Devitalization" OR "Dental Pulp Calcification" OR "Pulp Calcification" OR "necrotic pulp") |
| Web of Science | TS=("Sickle Cell Anemia" OR "Sickle Cell Anemias" OR "Sickle Cell Anaemia" OR "Sickle Cell Anaemias" OR  "Sickle Cell Disease" OR "Sickle Cell Diseases" OR "Sickle Cell Disorders" OR "Sickle Cell Disorder" OR "HbS Disease" OR "Sickle Hemoglobin" OR "Sickle cell Hemoglobin" OR "Hemoglobins"  OR "Haemoglobins" OR "Abnormal Hemoglobins" OR "Sickle Hemoglobin" OR "hemoglobin S" OR "haemoglobin S" OR "hemoglobin SC" OR "haemoglobin SC" OR "hemoglobin SE" OR "haemoglobin SE" OR "hemoglobin SS" OR "haemoglobin SS" OR "hemoglobin C disease" OR "hemoglobin D disease" OR "hemoglobin E disease" OR "haemoglobin C disease" OR "haemoglobin D disease" OR "haemoglobin E disease" OR "Hb SC" OR HbSC OR HbAS OR HbSS OR HbAC OR "Hb SE" OR "Hb SS" OR "Hb C disease" OR "Hb D disease" OR "Hb E disease" OR "SC disease" OR "SC diseases" OR "sickle cell*" OR sicklemia OR sickled OR sickling OR meniscocyt* OR drepanocyt* OR "Hemoglobin SC Disease" OR "Hemoglobin SC Diseases" OR "Sickle Cell Hemoglobin C Disease" OR "Sickle Cell Trait" OR "Sickle Cell Traits" OR Thalassemi* OR "alpha-Thalassemia" OR "beta-Thalassemia" OR "Mediterranean Anemia" OR "Erythrocyte Indices") AND TS=("Dental Pulp Necrosis" OR "Pulp Necrosis" OR "Pulpal Necrosis" OR "Pulpal Diagnosis of Necrosis" OR "Pulp Necroses" OR "Pulpal Necroses" OR "Pulpal Diagnosis of Necroses" OR "Pulp Mummification" OR "Pulp Mummifications" OR "Pulp Gangrene" OR "Pulp Gangrenes" OR "Dental Pulp" OR "Dental Pulps" OR "Dental Pulp Test" OR "Dental Pulp Diseases" OR "Pulp Diseases" OR "Dental Pulp Devitalization" OR "Pulp Devitalization" OR "Dental Pulp Calcification" OR "Pulp Calcification" OR "necrotic pulp") |
| Cochrane | ("Sickle Cell Anemia" OR "Sickle Cell Anemias" OR "Sickle Cell Anaemia" OR "Sickle Cell Anaemias" OR  "Sickle Cell Disease" OR "Sickle Cell Diseases" OR "Sickle Cell Disorders" OR "Sickle Cell Disorder" OR "HbS Disease" OR "Sickle Hemoglobin" OR "Sickle cell Hemoglobin" OR "Hemoglobins"  OR "Haemoglobins" OR "Abnormal Hemoglobins" OR "Sickle Hemoglobin" OR "hemoglobin S" OR "haemoglobin S" OR "hemoglobin SC" OR "haemoglobin SC" OR "hemoglobin SE" OR "haemoglobin SE" OR "hemoglobin SS" OR "haemoglobin SS" OR "hemoglobin C disease" OR "hemoglobin D disease" OR "hemoglobin E disease" OR "haemoglobin C disease" OR "haemoglobin D disease" OR "haemoglobin E disease" OR "Hb SC" OR HbSC OR HbAS OR HbSS OR HbAC OR "Hb SE" OR "Hb SS" OR "Hb C disease" OR "Hb D disease" OR "Hb E disease" OR "SC disease" OR "SC diseases" OR "sickle cell*" OR sicklemia OR sickled OR sickling OR meniscocyt* OR drepanocyt* OR "Hemoglobin SC Disease" OR "Hemoglobin SC Diseases" OR "Sickle Cell Hemoglobin C Disease" OR "Sickle Cell Trait" OR "Sickle Cell Traits" OR Thalassemi* OR "alpha-Thalassemia" OR "beta-Thalassemia" OR "Mediterranean Anemia" OR "Erythrocyte Indices") AND ("Dental Pulp Necrosis" OR "Pulp Necrosis" OR "Pulpal Necrosis" OR "Pulpal Diagnosis of Necrosis" OR "Pulp Necroses" OR "Pulpal Necroses" OR "Pulpal Diagnosis of Necroses" OR "Pulp Mummification" OR "Pulp Mummifications" OR "Pulp Gangrene" OR "Pulp Gangrenes" OR "Dental Pulp" OR "Dental Pulps" OR "Dental Pulp Test" OR "Dental Pulp Diseases" OR "Pulp Diseases" OR "Dental Pulp Devitalization" OR "Pulp Devitalization" OR "Dental Pulp Calcification" OR "Pulp Calcification" OR "necrotic pulp") |
| LILACS | ("sickle cell" OR "sickle cells" OR "HbS Disease" OR hemoglobin* OR haemoglobin* OR "Hb SC" OR "HbSC" OR "HbAS" OR "HbSS" OR "HbAC" OR "Hb SE" OR "Hb SS" OR "Hb C disease" OR "Hb D disease" OR "Hb E disease" OR "SC disease" OR "SC diseases" OR sicklemia OR sickled OR sickling OR meniscocyt* OR drepanocyt* OR thalassemi* OR "Mediterranean Anemia" OR "Erythrocyte Indices" OR falciforme* OR "Doença HbS" OR "doença da Hb C" OR "doença da Hb D" OR "doença de Hb E" OR "doenças SC" OR "falcemia" OR meniscócito* OR drepanócitos* OR "Anemia Mediterranea" OR talassemi* OR "Índices eritrocitários" OR "Enfermedad De HbS" OR "Enfermedad De La Hb C" OR "Enfermedad De La Hb D" OR " enfermedad Hb E" OR "Enfermedades SC" OR drepanocito* OR talasemi* OR "recuento de eritrocitos") AND ("Pulp Necrosis" OR "Pulpal Necrosis" OR "Pulpal Diagnosis of Necrosis" OR "Pulp Necroses" OR "Pulpal Necroses" OR "Pulpal Diagnosis of Necroses" OR "Pulp Mummification" OR "Pulp Mummifications" OR "Pulp Gangrene" OR "Pulp Gangrenes" OR "Dental Pulp" OR "Dental Pulps" OR "Pulp Diseases" OR "Pulp Devitalization" OR "Pulp Calcification" OR "necrotic pulp" OR "Necrose Pulpar" OR "Pulpar de Necrose" OR "Mumificação Pulpar" OR "Gangrena Pulpar" OR "Polpa Dentária" OR "Polpas Dentárias" OR "Doenças da Polpa" OR "Doença da Polpa" OR "Desvitalização da Polpa" OR "Calcificação pulpar" OR "polpa necrótica" OR "necroses pulpares" OR "Necrosis dental" OR "Necrosis pulpar" OR "Momificación pulpar" OR "Momificaciones pulpares" OR "Pulpa dental" OR "Enfermedades de la pulpa" OR "Desvitalización de la pulpa" OR "Calcificación de la pulpa" OR "pulpa necrótica" OR "calcificaciones en la pulpa") AND ( db:("LILACS")) |
| Google Scholar | ("Sickle Cell" OR “Hemoglobin” OR “thalassemia”) AND ("Dental Pulp" OR "Dental Pulps" OR "Pulp Necrosis" OR "Pulpal Necrosis" OR "Pulp Necroses" OR "Pulpal Necroses") |
| ProQuest | all("Sickle Cell Anemia" OR "Sickle Cell Anemias" OR "Sickle Cell Anaemia" OR "Sickle Cell Anaemias" OR  "Sickle Cell Disease" OR "Sickle Cell Diseases" OR "Sickle Cell Disorders" OR "Sickle Cell Disorder" OR "HbS Disease" OR "Sickle Hemoglobin" OR "Sickle cell Hemoglobin" OR "Hemoglobins"  OR "Haemoglobins" OR "Abnormal Hemoglobins" OR "Sickle Hemoglobin" OR "hemoglobin S" OR "haemoglobin S" OR "hemoglobin SC" OR "haemoglobin SC" OR "hemoglobin SE" OR "haemoglobin SE" OR "hemoglobin SS" OR "haemoglobin SS" OR "hemoglobin C disease" OR "hemoglobin D disease" OR "hemoglobin E disease" OR "haemoglobin C disease" OR "haemoglobin D disease" OR "haemoglobin E disease" OR "Hb SC" OR HbSC OR HbAS OR HbSS OR HbAC OR "Hb SE" OR "Hb SS" OR "Hb C disease" OR "Hb D disease" OR "Hb E disease" OR "SC disease" OR "SC diseases" OR "sickle cell" OR "sickle cells" OR sicklemia OR sickled OR sickling OR meniscocyt* OR drepanocyt* OR "Hemoglobin SC Disease" OR "Hemoglobin SC Diseases" OR "Sickle Cell Hemoglobin C Disease" OR "Sickle Cell Trait" OR "Sickle Cell Traits" OR Thalassemi* OR "alpha-Thalassemia" OR "beta-Thalassemia" OR "Mediterranean Anemia" OR "Erythrocyte Indices") AND all("Dental Pulp Necrosis" OR "Pulp Necrosis" OR "Pulpal Necrosis" OR "Pulpal Diagnosis of Necrosis" OR "Pulp Necroses" OR "Pulpal Necroses" OR "Pulpal Diagnosis of Necroses" OR "Pulp Mummification" OR "Pulp Mummifications" OR "Pulp Gangrene" OR "Pulp Gangrenes" OR "Dental Pulp" OR "Dental Pulps" OR "Dental Pulp Test" OR "Dental Pulp Diseases" OR "Pulp Diseases" OR "Dental Pulp Devitalization" OR "Pulp Devitalization" OR "Dental Pulp Calcification" OR "Pulp Calcification" OR "necrotic pulp") |
